# Supplementary material for: Resolving the full spectrum of human genome variation using Linked-Reads
Source: Genome Res. 2019 Apr;29(4):635–45. doi: 10.1101/gr.234443.118 (PMC6442396; doi:10.1101/gr.234443.118)
Supplement: Supplemental Material [file supp_gr.234443.118_Supplemental_longranger-2.2.zip › longranger-2.2.tar.bz2!/lr2.2-public/lib/stan/cmdstan/stan/doc/api/html/multi__normal__prec_8hpp_source.html]

Stan: src/stan/prob/distributions/multivariate/continuous/multi\_normal\_prec.hpp Source File


|  |  |
| --- | --- |
|  | Stan  2.5.0  probability, sampling & optimization |


- Main Page
- Related Pages
- Namespaces
- Classes
- Files

- File List
- File Members

All Classes Namespaces Files Functions Variables Typedefs Enumerations Enumerator Friends Macros Pages

- src
- stan
- prob
- distributions
- multivariate
- continuous

multi\_normal\_prec.hpp

Go to the documentation of this file.

1 #ifndef STAN\_\_PROB\_\_DISTRIBUTIONS\_\_MULTIVARIATE\_\_CONTINUOUS\_\_MULTI\_NORMAL\_PREC\_HPP

2 #define STAN\_\_PROB\_\_DISTRIBUTIONS\_\_MULTIVARIATE\_\_CONTINUOUS\_\_MULTI\_NORMAL\_PREC\_HPP

3

4 #include <stan/agrad/rev.hpp>

5 #include <stan/agrad/rev/matrix.hpp>

6 #include <stan/math/error\_handling.hpp>

7 #include <stan/math/matrix/columns\_dot\_product.hpp>

8 #include <stan/math/matrix/columns\_dot\_self.hpp>

9 #include <stan/math/matrix/dot\_product.hpp>

10 #include <stan/math/matrix/dot\_self.hpp>

11 #include <stan/math/matrix\_error\_handling.hpp>

12 #include <stan/math/matrix/log\_determinant\_ldlt.hpp>

13 #include <stan/math/matrix/log.hpp>

14 #include <stan/math/matrix/log\_determinant.hpp>

15 #include <stan/math/matrix/mdivide\_left\_spd.hpp>

16 #include <stan/math/matrix/mdivide\_left\_tri\_low.hpp>

17 #include <stan/math/matrix/multiply.hpp>

18 #include <stan/math/matrix/subtract.hpp>

19 #include <stan/math/matrix/sum.hpp>

20 #include <stan/math/matrix/trace\_quad\_form.hpp>

21 #include <stan/agrad/rev/matrix/trace\_quad\_form.hpp>

22 #include <stan/meta/traits.hpp>

23 #include <stan/prob/constants.hpp>

24 #include <stan/prob/traits.hpp>

25 #include <stan/math/error\_handling/matrix/check\_ldlt\_factor.hpp>

26

27 namespace stan {

28

29  namespace prob {

30

31  template <bool propto,

32  typename T\_y, typename T\_loc, typename T\_covar>

33  typename boost::math::tools::promote\_args<typename scalar\_type<T\_y>::type, typename scalar\_type<T\_loc>::type, T\_covar>::type

34  multi\_normal\_prec\_log(const T\_y& y,

35  const T\_loc& mu,

36  const Eigen::Matrix<T\_covar,Eigen::Dynamic,Eigen::Dynamic>& Sigma) {

37  static const char\* function = "stan::prob::multi\_normal\_prec\_log(%1%)";

38  typedef typename boost::math::tools::promote\_args<typename scalar\_type<T\_y>::type, typename scalar\_type<T\_loc>::type, T\_covar>::type lp\_type;

39  lp\_type lp(0.0);

40

41  using stan::math::check\_not\_nan;

42  using stan::math::check\_symmetric;

43  using stan::math::check\_size\_match;

44  using stan::math::check\_positive;

45  using stan::math::check\_finite;

46  using stan::math::sum;

47  using stan::math::trace\_quad\_form;

48  using stan::math::log\_determinant\_ldlt;

49  using stan::math::LDLT\_factor;

50  using stan::math::check\_ldlt\_factor;

51

52  check\_size\_match(function,

53  Sigma.rows(), "Rows of precision parameter",

54  Sigma.cols(), "columns of precision parameter",

55  &lp);

56  check\_positive(function, Sigma.rows(), "Precision matrix rows", &lp);

57  check\_symmetric(function, Sigma, "Precision matrix", &lp);

58

59  LDLT\_factor<T\_covar,Eigen::Dynamic,Eigen::Dynamic> ldlt\_Sigma(Sigma);

60  check\_ldlt\_factor(function,ldlt\_Sigma,

61  "LDLT\_Factor of precision parameter",&lp);

62

63  using Eigen::Matrix;

64  using Eigen::Dynamic;

65  using std::vector;

66  VectorViewMvt<const T\_y> y\_vec(y);

67  VectorViewMvt<const T\_loc> mu\_vec(mu);

68  //size of std::vector of Eigen vectors

69  size\_t size\_vec = max\_size\_mvt(y, mu);

70

71

72  //Check if every vector of the array has the same size

73  int size\_y = y\_vec[0].size();

74  int size\_mu = mu\_vec[0].size();

75  if (size\_vec > 1) {

76  int size\_y\_old = size\_y;

77  int size\_y\_new;

78  for (size\_t i = 1, size\_ = length\_mvt(y); i < size\_; i++) {

79  int size\_y\_new = y\_vec[i].size();

80  check\_size\_match(function,

81  size\_y\_new, "Size of one of the vectors of the random variable",

82  size\_y\_old, "Size of another vector of the random variable",

83  &lp);

84  size\_y\_old = size\_y\_new;

85  }

86  int size\_mu\_old = size\_mu;

87  int size\_mu\_new;

88  for (size\_t i = 1, size\_ = length\_mvt(mu); i < size\_; i++) {

89  int size\_mu\_new = mu\_vec[i].size();

90  check\_size\_match(function,

91  size\_mu\_new, "Size of one of the vectors of the location variable",

92  size\_mu\_old, "Size of another vector of the location variable",

93  &lp);

94  size\_mu\_old = size\_mu\_new;

95  }

96  (void) size\_y\_old;

97  (void) size\_y\_new;

98  (void) size\_mu\_old;

99  (void) size\_mu\_new;

100  }

101

102  check\_size\_match(function,

103  size\_y, "Size of random variable",

104  size\_mu, "size of location parameter",

105  &lp);

106  check\_size\_match(function,

107  size\_y, "Size of random variable",

108  Sigma.rows(), "rows of covariance parameter",

109  &lp);

110  check\_size\_match(function,

111  size\_y, "Size of random variable",

112  Sigma.cols(), "columns of covariance parameter",

113  &lp);

114

115  for (size\_t i = 0; i < size\_vec; i++) {

116  check\_finite(function, mu\_vec[i], "Location parameter", &lp);

117  check\_not\_nan(function, y\_vec[i], "Random variable", &lp);

118  }

119

120  if (size\_y == 0) //y\_vec[0].size() == 0

121  return lp;

122

123  if (include\_summand<propto,T\_covar>::value)

124  lp += 0.5 \* log\_determinant\_ldlt(ldlt\_Sigma) \* size\_vec;

125

126  if (include\_summand<propto>::value)

127  lp += NEG\_LOG\_SQRT\_TWO\_PI \* size\_y \* size\_vec;

128

129  if (include\_summand<propto,T\_y,T\_loc,T\_covar>::value) {

130  lp\_type sum\_lp\_vec(0.0);

131  for (size\_t i = 0; i < size\_vec; i++) {

132  Matrix<typename

133  boost::math::tools::promote\_args<typename scalar\_type<T\_y>::type, typename scalar\_type<T\_loc>::type>::type,

134  Dynamic, 1> y\_minus\_mu(size\_y);

135  for (int j = 0; j < size\_y; j++)

136  y\_minus\_mu(j) = y\_vec[i](j)-mu\_vec[i](j);

137  sum\_lp\_vec += trace\_quad\_form(Sigma,y\_minus\_mu);

138  }

139  lp -= 0.5\*sum\_lp\_vec;

140  }

141  return lp;

142  }

143

144  template <typename T\_y, typename T\_loc, typename T\_covar>

145  inline

146  typename boost::math::tools::promote\_args<typename scalar\_type<T\_y>::type, typename scalar\_type<T\_loc>::type, T\_covar>::type

147  multi\_normal\_prec\_log(const T\_y& y,

148  const T\_loc& mu,

149  const Eigen::Matrix<T\_covar,Eigen::Dynamic,Eigen::Dynamic>& Sigma) {

150  return multi\_normal\_prec\_log<false>(y,mu,Sigma);

151  }

152

153  }

154 }

155 #endif

156

columns\_dot\_self.hpp

matrix.hpp

mdivide\_left\_tri\_low.hpp

stan::max\_size\_mvt

size\_t max\_size\_mvt(const T1 &x1, const T2 &x2)

**Definition:** traits.hpp:575

traits.hpp

stan::prob::multi\_normal\_prec\_log

boost::math::tools::promote\_args< typename scalar\_type< T\_y >::type, typename scalar\_type< T\_loc >::type, T\_covar >::type multi\_normal\_prec\_log(const T\_y &y, const T\_loc &mu, const Eigen::Matrix< T\_covar, Eigen::Dynamic, Eigen::Dynamic > &Sigma)

**Definition:** multi\_normal\_prec.hpp:34

columns\_dot\_product.hpp

stan::math::check\_size\_match

bool check\_size\_match(const char \*function, T\_size1 i, const char \*name\_i, T\_size2 j, const char \*name\_j, T\_result \*result)

**Definition:** check\_size\_match.hpp:13

error\_handling.hpp

trace\_quad\_form.hpp

mdivide\_left\_spd.hpp

stan::math::check\_finite

bool check\_finite(const char \*function, const T\_y &y, const char \*name, T\_result \*result)

Checks if the variable y is finite.

**Definition:** check\_finite.hpp:52

log.hpp

stan::scalar\_type::type

scalar\_type\_helper< is\_vector< T >::value, T >::type type

**Definition:** traits.hpp:139

matrix\_error\_handling.hpp

constants.hpp

stan::agrad::trace\_quad\_form

stan::agrad::fvar< T > trace\_quad\_form(const Eigen::Matrix< stan::agrad::fvar< T >, RA, CA > &A, const Eigen::Matrix< stan::agrad::fvar< T >, RB, CB > &B)

**Definition:** trace\_quad\_form.hpp:19

traits.hpp

stan::math::check\_symmetric

bool check\_symmetric(const char \*function, const Eigen::Matrix< T\_y, Eigen::Dynamic, Eigen::Dynamic > &y, const char \*name, T\_result \*result)

Return true if the specified matrix is symmetric.

**Definition:** check\_symmetric.hpp:31

stan::math::check\_ldlt\_factor

bool check\_ldlt\_factor(const char \*function, stan::math::LDLT\_factor< T, R, C > &A, const char \*name, T\_result \*result)

Return true if the underlying matrix is positive definite.

**Definition:** check\_ldlt\_factor.hpp:24

dot\_self.hpp

check\_ldlt\_factor.hpp

stan::prob::include\_summand

Template metaprogram to calculate whether a summand needs to be included in a proportional (log) prob...

**Definition:** traits.hpp:35

rev.hpp

stan::math::LDLT\_factor

**Definition:** LDLT\_factor.hpp:16

log\_determinant\_ldlt.hpp

stan::math::trace\_quad\_form

double trace\_quad\_form(const Eigen::Matrix< double, RA, CA > &A, const Eigen::Matrix< double, RB, CB > &B)

Compute trace(B^T A B).

**Definition:** trace\_quad\_form.hpp:17

log\_determinant.hpp

stan::length\_mvt

size\_t length\_mvt(const T &)

**Definition:** traits.hpp:561

stan::math::check\_positive

bool check\_positive(const char \*function, const T\_y &y, const char \*name, T\_result \*result)

**Definition:** check\_positive.hpp:54

dot\_product.hpp

stan::math::sum

double sum(std::vector< double > &x)

**Definition:** sum.hpp:10

trace\_quad\_form.hpp

stan::math::check\_not\_nan

bool check\_not\_nan(const char \*function, const T\_y &y, const char \*name, T\_result \*result)

Checks if the variable y is nan.

**Definition:** check\_not\_nan.hpp:56

stan::agrad::log\_determinant\_ldlt

var log\_determinant\_ldlt(stan::math::LDLT\_factor< var, R, C > &A)

**Definition:** log\_determinant\_ldlt.hpp:48

size\_

size\_t size\_

**Definition:** dot\_self.hpp:18

stan::VectorViewMvt

**Definition:** traits.hpp:514

stan::math::log\_determinant\_ldlt

T log\_determinant\_ldlt(stan::math::LDLT\_factor< T, R, C > &A)

**Definition:** log\_determinant\_ldlt.hpp:12

subtract.hpp

sum.hpp

multiply.hpp


---

[ Stan Home Page ]

*© 2011–2014,
Stan Development Team.*
